# Physical Activity, Sedentary Behavior, and Quality of Life among University Students

**DOI:** 10.1155/2019/9791281

**Published:** 2019-12-18

**Authors:** Paweł F. Nowak, Agnieszka Bożek, Mateusz Blukacz

**Affiliations:** ^1^Faculty of Physical Education and Physiotherapy, Opole University of Technology, Opole, Poland; ^2^Institute of Psychology, Jagiellonian Univeristy, Cracow, Poland

## Abstract

The aim of our study was to explore the relation between physical activity, sedentary behavior, and the subjective and objective indicators of quality of life as well as life satisfaction among university students, whose education is related to different dimensions on health. Participants (*N* = 595) were invited to fill in a set of suitable questionnaires. The path analysis and linear regression were used to establish a relationship between the examined constructs. Only some types of physical activity have shown a positive relation with the quality of life; the study also revealed some age and gender regularities. Physical activity in the household was most positively correlated to the quality of life. The amount of leisure and transport physical activity decreased with age, and there were also gender differences regarding the intensity and type of physical activity. Sedentary behavior during the week related positively with the subjective quality of life and its intimacy dimension, but sedentary behavior at the weekends was negatively related to objective and subjective quality of life as well as dimensions including intimacy, safety, and communicative aspect of the quality of life. Neither physical activity nor sedentary behavior demonstrated a significant relation with the level of life satisfaction. The type of physical activity undertaken and its matching to the needs of the young person affected their objective and subjective quality of life. Those findings may have important implications for institutions responsible for promoting active lifestyle.

## 1. Introduction

Physical activity is commonly considered important, as the needs of a modern lifestyle can be fulfilled in this manner. It serves to fulfill the biologically conditioned need to undertake movement activities and forms one of the dimensions of everyday activity. It affects health and efficient everyday activity; it can also reduce health care costs [1]. However, beside the utilitarian aspects, physical activity is also a cultural manifestation of spending leisure time. The data from high-income countries confirm a decrease in human muscle work relating to performing professional duties; on the other hand, physical activity of a recreational nature is still increasing [2].

Physical activity is considered to be a positive health measure. It forms an integral part of a healthy lifestyle desired by society, especially in the western culture, as a healthy lifestyle is a determinant of health in all its dimensions [3]. Being considered as a healthy behavior, physical activity forms a significant element of the public health policy system in both developing and developed countries [4]. It is also a worldwide recognized health promotion tool, because it directly increases the health potential not only in the biological dimension of the organism but also in the psychosocial dimension. This is why the WHO disseminates information regarding spending human's energy through physical activity, with a certain volume and frequency, which is the biological minimum for the development of and keeping a good health [5]. However, in opposition to educational intervention and programs promoting active or sporty lifestyle, dissemination of sedentary culture (i.e., comfortable life and avoiding effort) can also be observed; this culture is determined by many factors related to development of modern civilization [6, 7].

It is worth emphasizing that one in five people in the world is completely physically inactive [8]. The amount of sedentary behavior grows with wealth of societies and the level of urban development. Inactivity is more common among women than men and this tendency increases with age. Up to one third of adults and four fifths of the youth do not achieve the recommended level of physical activity [2]. Researchers emphasize that the amount of time spent in front of computer screen or TV goes up systematically, and this has a negative impact on quality of life related to health, combined with lack of physical activity [9]. Additionally, the lack of physical activity is the fourth leading cause of deaths in the world [10].

Contemporary strategies disseminating physical activity and research concepts in this area focus on several key areas, where an individual can spend their energy using their own muscles. Four types of physical activity are distinguished: activity related to professional work, activity related to movement throughout the day, activity related to household duties, and recreational activity performed in the leisure time [11]. An examination regarding the level of physical activity in the group on the emerging adults is particularly important, as in this period of life, health habits are built up; it is also the period of diverse experiences that affect the way of life in the future [12].

Concept of quality of life has become widespread in the perspective of socioeconomic and cultural development [13]. It is a multidimensional construct; different studies take into account its different aspects. Previous studies have shown a positive relationship between physical activity and various indicators of the quality of life, but they mainly concerned older adults [14, 15] or chronically ill individuals [16]; most of them concerned only health-related quality of life [17, 18]. However, the present study focuses on both objective and subjective indicators of the quality of life and their association with physical activity or sedentary behavior among university students.

Life satisfaction deals with overall assessment of one's own achievements and living [19]. This is the degree to which a person evaluates the overall quality of their life—overall positively or negatively [20]. Results of different research studies are inconsistent, although most of them indicate a positive relation between physical activity as such and life satisfaction. For example, Maher and colleagues [21] found that college students who engaged in more physical activity that is typical for them experienced greater life satisfaction.

The purpose of the study is to examine the relationships between physical activity, sedentary behavior, and subjective and objective indicators of quality of life as well as life satisfaction among a group of university students, whose future work deals with either the human's body or mind. Based on current research [17, 22–26], we assumed that physical activity affects an increase in different indicators of quality of life and thereby increases the life satisfaction. Conversely, it was assumed that sedentary behavior contributes to a decrease in the quality of various aspects of the quality of life. The authors also wanted to find out the types of physical activity (i.e., activity associated with: professional work, household duties, recreation, or transport) and to what extent they relate to the indicators of quality of life and general life satisfaction.

## 2. Materials and Methods

### 2.1. Participants

The study was conducted among 595 students from six Polish universities. They were classified into two groups depending on the study course: 295 students of physical health, physiotherapy, and tourism and recreation, whose education is focused on the human body, and 300 students of psychology, pedagogy, or theology, whose education deals with the human mind and spirit. In total, there were 387 (65%) females and 208 (35%) males, aged from 18 to 30 (*M* = 21.67; SD = 1.88).

### 2.2. Measurements and Data Analysis

The authors applied the International Physical Activity Questionnaire [27] with the purpose of assessing physical activity undertaken across a comprehensive set of domains including leisure time physical activity; domestic and gardening activities; work-related physical activity; transport-related physical activity; and sedentary behavior measured separately during the week and at the weekend. The questionnaire contains 26 questions regarding the duration of time (expressed in minutes) that participants spend on the given activities.

To assess quality of life the authors used Comprehensive Quality of Life Scale—Adult (ComQol-A5) by Cummins [28]. This scale comprises 14 items and measures both objective and subjective dimensions of quality of life that cover seven areas: material well-being, health, productivity, intimacy, safety, place in community, and emotional well-being. The measurement of each objective domain is achieved by obtaining an aggregate importance score based on the measurement of three objective indices relevant to that domain. The measurement of each subjective domain is achieved by obtaining a satisfaction score of that domain, whose relevance is weighed by the perceived importance of the domain for an individual.

Life satisfaction was measured with the Satisfaction with Life Scale (SWLS) by Diener et al. [19]. The scale contains 5 items relating to the sense of satisfaction with one's own achievements and living conditions.

The study was carried out in groups of students. After obtaining permission to conduct research from study heads, participants completed a paper set of questionnaires in the presence of the investigator. Due to large data gaps or inadequate completion of the questionnaires (e.g., in the form of marking only the extreme answers), approximately 3% of the questionnaires were rejected.

The relations between quality of life and physical activity were modeled using path analysis, based on which a model was created so as to represent the structure of relations between various aspects of the analyzed constructs. The model is based on an assumption that the subjective quality of life depends on the amount of physical activity, but the relations are analyzed as different physical activities and separate domains representing the quality of life, while controlling for energy expenditure, age, gender, and type of education. This allowed the authors to identify and analyze in detail the relations often described in the literature as a single parameter. It is important to emphasize here that because of methodological limitations, since the research was neither experimental nor longitudinal, the presented model does not prove the causal effect of physical activity on quality of life. However, the focus of the present study is on the quality of life by quantifying the size and directions of the relations between the aspects of both constructs.

**Figure 1:**
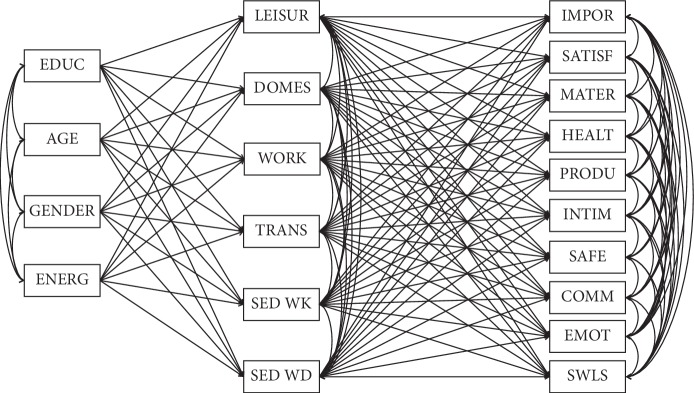
Structure of relations between variables tested in the model. EDUC: type of education; AGE: age; GENDER: gender; ENERG: weekly energy expenditure; LEISUR: leisure time physical activity; DOMES: domestic and gardening activities; WORK: work-related physical activity; TRANS: transport-related physical activity; SED WK: sedentary behavior during the week; SED WD: sedentary behavior during the weekend; IMPOR: importance score; SATISF: satisfaction score; MATER: material well-being; HEALTH: health; PRODU: productivity; INTIM: intimacy; SAFE: safety; COMM: place in community; EMOT: emotional well-being; SWLS: life satisfaction.

The model consists of three groups of variables: the principal two represent the aspects of physical activity and domains of quality of life, and control variables providing information such as the type of education, age, gender, and weekly energy expenditure. The aim was to assess the magnitude of possible relations between activity and quality of life, while adjusting for other factors that could affect them. To grasp the complexity of relationships, it was imperative to include life satisfaction in the model, a characteristic somewhat similar and correlated to quality of life. This allowed us to contrast whether physical activity is more inclined to predict quality of life or life satisfaction, while also controlling for weekly energetic expenditure [29]. The type of education was coded “0” for education related to the physical health and “1” for education focused on the mind and spirit. Gender was marked as “0” for men and “1” for women. The structure of tested model is presented in Figure 1.

## 3. Results

The analysis was conducted in Mplus 7 software [30].  Table 1 contains a summary of the statistics. Due to skewed distributions, physical activity time measures were analyzed as logarithms. All measures except some aspects of physical activity were gathered from 595 participants. The measure of work-related activity had the greatest number of the missing answers; yet since all other information about physical activities were collected, it was probably because some students were not employed at all. The total of the data that was missing in the questionnaires regarding work-related activity was not related to the type of education (*χ*^2^ (1) = 1.889 *p*=0.169) which allowed the assumption that the fact of finding missing data was in fact random, and thus, full information maximum likelihood estimation was used.

**Figure 2:**
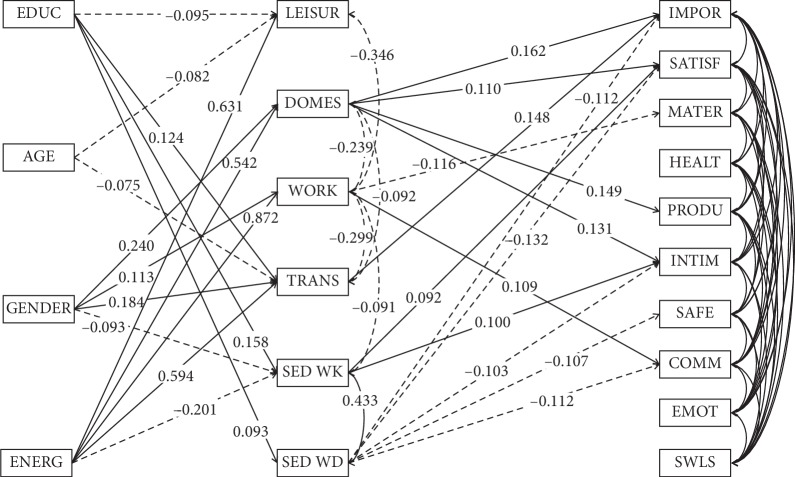
Standardized values of significant paths in the model. Abbreviations: EDUC: type of education; AGE: age; GENDER: gender; ENERG: weekly energy expenditure; LEISUR: leisure time physical activity; DOMES: domestic and gardening activities; WORK: work-related physical activity; TRANS: transport-related physical activity; SED WK: sedentary behavior during the week; SED WD: sedentary behavior during the weekend; IMPOR: importance score; SATISF: satisfaction score; MATER: material well-being; HEALTH: health; PRODU: productivity; INTIM: intimacy; SAFE: safety; COMM: place in community; EMOT: emotional well-being; SWLS: life satisfaction.

The estimated model is presented in Figure 2. For the purpose of clear illustration, nonsignificant paths were excluded from the graph. The fit indices show that the model is rather good fitting to the data (*χ*^2^/df = 90.881/40 = 2.272; RMSEA = 0.046; CFI = 0.990; TLI = 0.954) and was not modified from the model presented in Figure 1. The complete variance-covariance matrix is given in Table 2.

Paths without established materiality level were omitted from the graph (see Figure 1.). Solid lines represent positive relations; dashed lines represent negative relations. Correlation coefficients between domains of quality of life and demographic variables were also omitted from the graph.

The path analysis model offers the possibility to distinguish direct paths which are equivalent to regression coefficients and indirect effects which are paths that run from one variable to another through other variables. The total indirect effects are sums of all possible indirect paths that follow from one variable to another. Total indirect effects were tested for significance using Sobel's test [31]. Direct paths and total indirect effects, in unstandardized and standardized form, and correlation coefficients are presented in Table 3.

The model demonstrates that the education focusing on mind and spirit is related to less time being spent by participants on leisure activities (*p*=0.006) and a greater amount of time spent on transport (*p*=0.001) and sedentary activities both during the week and at the weekend (*p* < 0.001, *p*=0.035). Along with older age, participants are likely to spend less time on leisure (*p*=0.014) and transport activities (*p*=0.035). Interestingly, women seemed to spend more time on domestic (*p* < 0.001), work-related (*p* < 0.001), and transport activities (*p* < 0.001) and less time on sedentary activities during the week (*p*=0.022). Weekly energy expenditure depends on amount of time spent actively per week; thus, it is strongly related to all activity measures (*p* < 0.001), except for sedentary weekly activities which are negatively related to energy expenditure (*p* < 0.001). Work seems to have a higher energy expenditure.

The time spent on leisure was negatively correlated with time related to work (*r* = −0.346) which suggests that these activities form an alternative activity in the student group. Similar effects but with smaller magnitudes can be observed between work-related and domestic (*r* = −0.239), transport (*r* = −0.299), and sedentary weekly (*r* = −0.091) activities. The time spent on domestic activities is also negatively correlated with transport activities (*r* = −0.092). Sedentary weekly and weekend activities are positively correlated with each other (*r* = 0.433).

Domestic activity positively relates to the importance score (*p* < 0.001), satisfaction score (*p*=0.017), and productivity (*p*=0.001) and intimacy (*p*=0.004) domains of the quality of life. Work-related activities negatively relate to material quality of life (*p*=0.025) and positively to communicative quality of life (*p*=0.033). Transport activities seem to positively relate to importance score of quality of life (*p*=0.001). Sedentary weekly activities are positively related to satisfaction score (*p*=0.047) and intimacy domain of the quality of life (*p*=0.030). In contrast, sedentary weekend activities negatively relate to importance score (*p*=0.011), satisfaction score (*p*=0.004), intimacy (*p*=0.023), safety (*p*=0.018), and communication (*p*=0.014) domains of the quality of life.

## 4. Discussion

The aim of the study was to establish a relation between physical activity, sedentary behavior, and the subjective and objective indicators of quality of life as well as life satisfaction among university students, whose future work is concerned with either the human's body or mind. The results that were obtained confirm the starting assumptions only to a certain degree.

First, only some types of physical activity demonstrate a positive relation with the quality of life. Activities accompanying household duties have shown the greatest number of positive associations (i.e., with regard to the objective and subjective quality of life and productivity and intimacy dimensions of the quality of life). The activity during professional work was also established to be a significant factor (for the communication dimension of quality of life) along with the activities during transportation (for the objective quality of life). Surprisingly, no relationship could be established (neither positive nor negative) between the leisure time activity and the quality of life. Activity during leisure time is associated with greater energy expenditure, which leads to tiredness. Not everyone has positive experiences related to physical activity. Some people can associate it with a big effort, rivalry, hard work on oneself, self-discipline, and sacrifices, but not with pleasure.

Physical activity is a culturally conditioned behavior [32]. In recent years in Poland, where the study took place, a dynamic development of different forms of health-oriented physical recreation is observed [33]. However, traditions in this area are much more limited than in Western European countries [34, 35]. People still think that physical activities involving domestic work (e.g., cleaning and ironing) and transport (e.g., walking to work and climbing the stairs) can fulfil the biologically conditioned drive to perform movement and can substitute recreational sports in the leisure time. The same applies to physical work performed within the profession. Professional physical activity (e.g., work on site, in the factory, and in the bar) overloads the body in a one-sided manner, so it cannot be classified as a positive element of a healthy lifestyle. Research shows that engaging a physical work in one's profession may significantly reduce the physical activity in leisure time [36].

Second, the results of this study have demonstrated some demographic regularities related to physical activity. Women engage in physical domestic activity, work-related physical activity and transportation activity more frequently compared to men, and they spend less time than men in a sitting position. The result can be explained in terms of cultural customs, as in the Polish society, women are expected to take care of the household and children, do shopping, etc., as well as work professionally for at least part of the time. This, in turn, limits the time they can spend in a sitting position.

Third, the results reveal that the older participants were the less likely to spend time on physical activity during their leisure time. We can interpret it in such a way that the number of duties increases in the adult life (i.e., more time is needed for work or family) and the amount of free time decreases, and there is a smaller pressure from peers to spend free time in a physically active way. In addition, with age, the financial situation improves, so that more people may be able to afford to buy a vehicle instead of using a bike.

Fourth, the study indicates that students in mental and spiritual health-related study fields (such as psychology, pedagogy, and theology) are less interested in physical activities during their leisure time than students in physical health-related study fields (such as physical education and physiotherapy); the research shows that they would rather prefer spending their free time in a passive manner. However, this group reported more physical activity related to transport compared to the students from the latter group. This can be explained by the fact that people engaged in sports activities in their leisure time, e.g., rarely ride a bike or walk on foot, because they might think that their activity is performed by practicing their favorite sport or form of recreation [37]. As a result, they are able to select either to use a vehicle more often, which does not require the use of their muscles. The research also shows that physical activity is considered in an instrumental way and individual types of activity are used interchangeably, although they have different goals. Types of physical activity are negatively correlated to each other, which means that participants experience the energy expenditure associated with professional physical work or household duties and are less willing to take recreational physical activity.

Fifth, sedentary behaviors during the week usually have an impact on the way of spending leisure time on weekends [38]; the present research confirms this regularity since there is a significant relationship between time spent in a sitting position throughout a week and during weekends. The assumption about negative relationship between the sedentary behavior at the weekend on different dimensions of quality of life is confirmed in this study as well. People certainly perceive differently their activity inactivity during work days and at the weekend. Days off are typical for leisure and different organizations of the day. Lack of activity on weekdays fulfills the role of necessary rest and therefore can be a source of pleasure. Weekends are also treated as a chance to get satisfying entertainment, to deepen social contacts, etc. Probably, the responders were aware that physical activity would be a much better way to develop social contacts than sedentary behavior, hence the negative relationship of the latter with the dimensions of the quality of life.

The more surprising result was that the sedentary weekly behavior is positively related to subjective quality of life and its intimacy dimensions. It might be that sedentary behavior during the week plays some important role on the quality of life when it is balanced with other physical activities, and sedentary behavior during the weekend does not have a similar effect.

Another unexpected result of the study is that no significant relationship was established between physical activity and the overall life satisfaction. The reason for this may arise from the fact that the physical activity performed by the participants is not enjoyable for them, as, for example, its type and intensity does not suit them, and consequently, it is considered as a form of an unpleasant obligation. However, the quality of physical education classes attended by the respondents was not researched in this study. It is also not known in what kind of physical activity participants take part in their leisure time or whether it is tailored to their needs and capabilities; perhaps, this fact affects the results of the present study.

### 4.1. Limitations of the Study

The research has involved a fairly diverse group of students (different colleges and universities in different parts of the country), and differences could be established between the particular aspects of quality of life as well as sedentary behavior during week and weekends, thus allowing a comprehensive analysis of the relations. However, the present study is not free from limitations. As it was cross-sectional study and subjects were not randomized between the groups, the observed effects were not controlled for other between-group factors. Moreover, the methods used in the study only applied self-report measures. Thus, the participants' answers could be affected by memory biases as well as the response bias to fit in the social approval. Additionally, the study was conducted only in Poland, so some of the registered results could be hard to generalize or replicate due to cultural factors. Therefore, an approach involving a more robust comparison in terms of cultural and socioeconomic factors is recommended.

### 4.2. Implications

The study could present some important implications for college and universities as well as institutions responsible for promoting active lifestyle. Spending leisure time actively is a value in itself, as it contributes to the personal development of a person not only physically, but also has an impact on health in the psychosocial dimension [39].

The promotion of physical activity is one of the significant elements of the strategy of public health institutions [40]. In the consideration of obtained results, special attention should be paid to promoting a wide range of forms of recreational activity undertaken in the leisure time [41].

## 5. Conclusions

In the presented study of university students, whose future work should involve either the development of the human's body or mind, the study demonstrated that

- Physical activity relates significantly with the quality of life, however not all its types. Physical activity in the household is most positively correlated to the quality of life.
- The amount of leisure and transport physical activity decreases with age, and there are gender differences regarding the intensity and type of physical activity.
- Sedentary behavior during the week relates positively with the subjective quality of life and its intimacy dimension, but sedentary behavior at the weekends is negatively related to objective and subjective quality of life as well as dimensions including intimacy, safety, and communicative aspect of the quality of life.
- Neither physical activity nor sedentary behavior demonstrates a considerable relation with the level of life satisfaction.
- Physical activity has to be spread among students in such a manner that recreational activities of the secured quality are not substituted by household or professional duties or transport activities.

## Figures and Tables

**Table 1:** Descriptive statistics.

Variable		*N*	Mean or %	SD	Min	Max
Demographic	Age	595	21.73	1.99	18.00	30.00
	Gender (% female)	595	64.71%			
Education	Human body	295	49.58%			
	Human mind and spirit	300	50.42%			
Quality of life	Importance	595	4.11	0.48	2.14	5.00
	Satisfaction	595	5.20	0.83	1.00	7.00
	Material	595	6.33	5.58	−20.00	20.00
	Health	595	8.98	8.04	−20.00	20.00
	Productivity	595	8.05	6.64	−20.00	20.00
	Intimacy	595	12.51	7.23	−20.00	20.00
	Safety	595	10.66	6.75	−20.00	20.00
	Community	595	5.65	7.07	−20.00	20.00
	Emotional	595	9.63	8.38	−20.00	20.00
Well-being	SWLS	595	21.34	4.89	5.00	35.00
		*N*	Median	IQR	Min	Max
Physical activity (minutes per week)	Leisure time	592	1668.00	3274.50	0.00	32100.00
	Domestic and gardening	592	1200.00	1920.00	0.00	18660.00
	Work-related	413	5436.00	9126.00	33.00	49560.00
	Transport-related	589	1485.00	2466.00	49.50	20790.00
	Sedentary weekly	592	300.00	240.00	30.00	900.00
	Sedentary weekend	592	300.00	240.00	30.00	900.00
	Weekly energy expenditure (kcal × week−1)	592	9746.35	13398.29	323.30	67284.00
Model fit		*χ* ^2^	df	RMSEA	CFI	TLI
		90.881	40	0.046	0.990	0.954

Abbreviations: *N*: number of complete cases; df: degrees of freedom; IQR: interquartile; RMSEA: root means square error of approximation; CFI: comparative fit index; TLI: Tucker Lewis index.

**Table 2:** 

Variance-covariance matrix (*N* = 595)	LEISUR	DOMES	WORK	TRANS	SED-WK	SED-WD	IMPOR	SATISF	MATER	HEALT	PRODU	INTIM	SAFE	COMM	EMOT	SWLS	EDUC	AGE	GENDER	ENERG
LEISUR	1.581																			
DOMES	0.389	1.422																		
WORK	0.758	0.590	1.912																	
TRANS	0.418	0.319	0.528	1.160																
SED A	−0.104	−0.080	−0.185	−0.085	0.305															
SED B	−0.074	−0.030	−0.064	−0.049	0.134	0.293														
IMPOR	0.063	0.114	0.071	0.103	−0.031	−0.038	0.234													
SATISF	0.050	0.122	0.074	0.071	0.003	−0.046	0.174	0.687												
MATER	−0.195	0.304	−0.631	0.092	0.104	−0.030	0.792	2.295	31.047											
HEALT	0.056	0.394	0.396	0.319	−0.332	−0.414	1.182	4.178	11.243	64.526										
PRODU	0.557	1.258	0.618	0.542	−0.110	−0.274	1.391	4.117	12.748	22.418	44.043									
INTIM	0.278	1.214	0.613	0.722	0.107	−0.269	1.330	4.087	10.384	16.877	20.656	52.199								
SAFE	0.781	0.946	0.762	0.772	−0.063	−0.346	1.568	3.965	9.582	21.095	21.804	28.536	45.552							
COMM	0.252	0.635	1.067	0.811	−0.010	−0.334	1.212	3.197	6.311	11.738	18.879	12.218	12.940	49.935						
EMOT	0.406	0.952	1.027	0.578	0.072	−0.246	1.415	5.085	14.399	25.445	28.570	29.923	27.637	17.176	70.044					
SWLS	0.482	0.249	0.299	0.253	0.028	−0.122	0.681	2.323	8.014	13.228	16.256	13.456	13.053	8.330	21.258	23.835				
EDUC	−0.169	−0.046	−0.134	0.002	0.048	0.030	0.000	−0.002	0.083	−0.330	0.130	−0.045	−0.108	0.334	−0.323	0.061	0.250			
AGE	−0.268	0.136	−0.013	−0.092	0.000	0.049	0.009	−0.038	−0.428	−0.407	0.103	−1.270	−0.681	1.522	−0.464	−0.572	0.287	3.966		
GENDER	−0.112	0.078	−0.030	0.053	−0.007	−0.001	0.049	0.018	0.096	0.103	0.228	0.479	0.201	−0.030	0.275	0.145	0.043	−0.020	0.227	
ENERG	0.808	0.583	1.150	0.559	−0.118	−0.049	0.069	0.081	−0.273	0.342	0.546	0.569	0.834	0.903	0.738	0.316	−0.107	0.001	−0.082	0.943

**Table 3:** Unstandardized and standardized path coefficients: direct, total indirect, and correlation coefficients.

		Physical activity (log)							Quality of Life										Well-being	
			Leisur	Domes	Work	Trans	Sed Wk	Sed Wd		Impor	Satisf	Mater	Healt	Produ	Intim	Safe	Comm	Emot	SWLS	
		Direct effects							Total indirect										Total indirect	
Control variables	Education	*b*	−0.239	−0.047	−0.069	0.267	0.175	0.101	*b*	0.071	0.061	0.151	0.356	0.569	0.654	0.448	0.518	0.493	*b*	0.047
		*β*	−0.095	−0.020	−0.025	0.124	0.158	0.093	*β*	0.070	0.035	0.013	0.021	0.041	0.043	0.032	0.035	0.028	*β*	0.005
		*p*	0.006	0.603	0.335	0.001	<0.001	0.035	*p*	<0.001	0.020	0.378	0.143	0.006	0.004	0.030	0.017	0.050	*p*	0.744
	Age	*b*	−0.052	0.041	0.002	−0.041	−0.013	0.005	*b*	0.000	0.010	0.109	−0.133	−0.032	0.206	0.025	0.191	0.125	*b*	−0.005
		*β*	−0.082	0.069	0.003	−0.075	−0.047	0.018	*β*	0.000	0.006	0.010	−0.008	−0.002	0.014	0.002	0.014	0.007	*β*	−0.001
		*p*	0.014	0.067	0.898	0.032	0.254	0.674	*p*	0.988	0.634	0.395	0.460	0.840	0.249	0.876	0.254	0.508	*p*	0.963
	Gender	*b*	−0.153	0.602	0.331	0.415	−0.108	−0.039	*b*	0.000	−0.001	0.003	0.014	0.013	0.001	−0.022	−0.015	−0.002	*b*	−0.021
		*β*	−0.058	0.240	0.113	0.184	−0.093	−0.034	*β*	−0.001	−0.002	0.001	0.003	0.004	0.000	−0.006	−0.004	0.000	*β*	−0.009
		*p*	0.077	<0.001	<0.001	<0.001	0.022	0.417	*p*	0.911	0.867	0.903	0.675	0.668	0.967	0.458	0.627	0.952	*p*	0.250
	Weekly	*b*	0.817	0.667	1.248	0.659	−0.114	−0.044	*b*	0.089	0.087	−0.242	0.345	0.714	0.767	0.902	0.880	0.891	*b*	0.352
	Energy	*β*	0.631	0.542	0.872	0.594	−0.201	−0.079	*β*	0.179	0.102	−0.042	0.042	0.104	0.103	0.130	0.121	0.103	*β*	0.070
	Expenditure	*p*	<0.001	<0.001	<0.001	<0.001	<0.001	0.060	*p*	<0.001	0.011	0.295	0.300	0.009	0.010	0.001	0.002	0.010	*p*	0.081

		Correlations							Direct effects										Direct effects	
Physical activity (log)	Leisure	*r*	—	—	—	—	—	—	*b*	0.002	−0.007	−0.045	−0.196	0.059	−0.194	0.195	−0.294	−0.140	*b*	0.227
		*p*	—	—	—	—	—	—	*β*	0.006	−0.010	−0.010	−0.031	0.011	−0.034	0.036	−0.052	−0.021	*β*	0.058
									*p*	0.895	0.830	0.834	0.526	0.818	0.482	0.451	0.275	0.663	*p*	0.234
	Domestic	*r*	−0.086	—	—	—	—	—	*b*	0.066	0.076	0.387	0.211	0.826	0.794	0.496	0.226	0.499	*b*	0.088
		*p*	0.061	—	—	—	—	—	*β*	0.162	0.110	0.083	0.031	0.149	0.131	0.088	0.038	0.071	*β*	0.022
									*p*	<0.001	0.017	0.073	0.500	0.001	0.004	0.056	0.399	0.116	*p*	0.637
	Work	*r*	−0.346	−0.239	—	—	—	—	*b*	−0.006	0.021	−0.464	0.132	−0.022	0.157	0.148	0.555	0.489	*b*	0.061
		*p*	<0.001	<0.001	—	—	—	—	*β*	−0.018	0.035	−0.116	0.023	−0.005	0.030	0.030	0.109	0.081	*β*	0.017
									*p*	0.727	0.498	0.025	0.660	0.927	0.560	0.557	0.033	0.117	*p*	0.738
	Transport	*r*	−0.044	−0.092	−0.299	—	—	—	*b*	0.066	0.035	0.213	0.143	0.211	0.438	0.390	0.503	0.223	*b*	0.098
		*p*	0.313	0.029	<0.001	—	—	—	*β*	0.148	0.045	0.041	0.019	0.034	0.065	0.062	0.077	0.029	*β*	0.022
									*p*	0.001	0.313	0.360	0.669	0.442	0.141	0.161	0.085	0.522	*p*	0.631
	Sed weekly	*r*	0.004	0.010	−0.091	−0.036	—	—	*b*	−0.024	0.138	0.329	−0.464	0.341	1.313	0.772	1.043	1.218	*b*	0.496
		*p*	0.930	0.820	0.048	0.381	—	—	*β*	−0.027	0.092	0.033	−0.032	0.028	0.100	0.063	0.082	0.080	*β*	0.056
									*p*	0.550	0.047	0.486	0.496	0.541	0.030	0.173	0.079	0.085	*p*	0.232
	Sed weekend	*r*	−0.048	0.005	−0.005	−0.046	0.433	—	*b*	−0.100	−0.202	−0.291	−1.173	−0.960	−1.373	−1.334	−1.458	−1.231	*b*	−0.547
		*p*	0.284	0.909	0.922	0.262	<0.001	—	*β*	−0.112	−0.132	−0.028	−0.079	−0.078	−0.103	−0.107	−0.112	−0.080	*β*	−0.061
									*p*	0.011	0.004	0.536	0.083	0.083	0.023	0.018	0.014	0.080	*p*	0.184

									Correlations											
Quality of life	Importance		—	—	—	—	—	—	*r*	—	—	—	—	—	—	—	—	—	—	—
			—	—	—	—	—	—	*p*	—	—	—	—	—	—	—	—	—	—	—
	Satisfaction		—	—	—	—	—	—	*r*	0.414	—	—	—	—	—	—	—	—	—	—
			—	—	—	—	—	—	*p*	<0.001	—	—	—	—	—	—	—	—	—	—
	Material		—	—	—	—	—	—	*R*	0.297	0.501	—	—	—	—	—	—	—	—	—
			—	—	—	—	—	—	*p*	<0.001	<0.001	—	—	—	—	—	—	—	—	—
	Health		—	—	—	—	—	—	*r*	0.293	0.628	0.254	—	—	—	—	—	—	—	—
			—	—	—	—	—	—	*p*	<0.001	<0.001	<0.001	—	—	—	—	—	—	—	—
	Productivity		—	—	—	—	—	—	*r*	0.411	0.743	0.344	0.417	—	—	—	—	—	—	—
			—	—	—	—	—	—	*p*	<0.001	<0.001	<0.001	<0.001	—	—	—	—	—	—	—
	Intimacy		—	—	—	—	—	—	*r*	0.358	0.672	0.254	0.286	0.414	—	—	—	—	—	—
			—	—	—	—	—	—	*p*	<0.001	<0.001	<0.001	<0.001	<0.001	—	—	—	—	—	—
	Safety		—	—	—	—	—	—	*r*	0.461	0.700	0.256	0.385	0.473	0.574	—	—	—	—	—
			—	—	—	—	—	—	*p*	<0.001	<0.001	<0.001	<0.001	<0.001	<0.001	—	—	—	—	—
	Community		—	—	—	—	—	—	*r*	0.337	0.535	0.165	0.198	0.393	0.218	0.252	—	—	—	—
			—	—	—	—	—	—	*p*	<0.001	<0.001	<0.001	<0.001	<0.001	<0.001	<0.001	—	—	—	—
	Emotional		—	—	—	—	—	—	*r*	0.337	0.728	0.313	0.376	0.507	0.483	0.479	0.273	—	—	—
			—	—	—	—	—	—	*p*	<0.001	<0.001	<0.001	<0.001	<0.001	<0.001	<0.001	<0.001	—	—	—
Well-being	SWLS		—	—	—	—	—	—	*r*	0.283	0.572	0.298	0.337	0.500	0.377	0.388	0.234	0.234	—	—
			—	—	—	—	—	—	*p*	<0.001	<0.001	<0.001	<0.001	<0.001	<0.001	<0.001	<0.001	<0.001	—	—

## Data Availability

The data used to support the findings of this study are available from the corresponding author upon request.
